# Aqueous/Aqueous Micro Phase Separation: Construction of an Artificial Model of Cellular Assembly

**DOI:** 10.3389/fchem.2019.00044

**Published:** 2019-02-01

**Authors:** Hiroki Sakuta, Tadashi Fujimoto, Yusuke Yamana, Yusuke Hoda, Kanta Tsumoto, Kenichi Yoshikawa

**Affiliations:** ^1^Graduate School of Life and Medical Sciences, Doshisha University, Kyoto, Japan; ^2^Division of Chemistry for Materials, Graduate School of Engineering, Mie University, Tsu, Japan

**Keywords:** aqueous two-phase system, microdroplet, phase separation, cell assembly, red blood cell, epithelial cell, polyethylene glycol, dextran

## Abstract

To artificially construct a three-dimensional cell assembly, we investigated the availability of long-duration microdroplets that emerged near a critical point in an aqueous two-phase system (ATPS) with the hydrophilic binary polymers, polyethylene glycol (PEG), and dextran (DEX), as host containers. We found that erythrocytes (horse red blood cells; RBCs) and NAMRU mouse mammary gland epithelial cells (NMuMG cells) were completely and spontaneously entrapped inside DEX-rich microdroplets. RBCs and NMuMG cells were located in the interior and at the periphery of the droplets at PEG/DEX = 5%:5%. In contrast, the cells exhibited opposite localizations at PEG/DEX = 10%:5%, where, interestingly, NMuMG cells apparently assembled to achieve cell adhesion. We simply interpreted such specific localizations by considering the alternative responses of these cells to the properties of the PEG/DEX interfaces with different gradients in polymer concentrations.

## Introduction

Living organisms exhibit the self-organization of cells and intracellular organelles in a self-consistent manner. For decades, biochemical studies have unveiled the mechanisms that underlie such regulatory systems at the molecular level. Many macromolecules including nucleic acids and proteins are considered to act in a well-orchestrated manner to express biological functions, by building sophisticated structures under the specific interaction of gene products. On the other hand, it is also considered that even a single-cell system cannot be completely controlled through specific key-lock interactions. In these situations, the self-organization, or self-assembly, of biomolecules is indispensable for the development of various ordered structures, like phospholipid bilayers for cell membranes. Recently, it has been reported that membraneless organelles may arise from the micro phase separation of cytosols, which generates submicron-scale liquid droplets containing proteins and RNAs (Brangwynne, 2013; Courchaine et al., 2016). Since intracellular environments are highly crowded by such macromolecules, the emergence of liquid/liquid phase separation (LLPS) on a microscopic scale is a ubiquitous phenomenon inside living cells (Walter and Brooks, 1995; Spitzer and Poolman, 2013; Rivas and Minton, 2016; Uversky, 2017).

Along these lines, many pioneering researchers have tried to model cellular and intracellular microcompartments by simply using an aqueous two-phase system (ATPS) consisting of solutions with hydrophilic polymers that exhibit LLPS on a micro-scale (Aumiller and Keating, 2017). There are both some similarities and differences in the properties of micro phase separation between *in vitro* ATPS droplets and actual protein/nucleic acid-based droplets (Zaslavsky et al., 2018). Polyethylene glycol (PEG), a flexible polymer, and dextran (DEX), a semi-flexible polymer, are popularly selected for the preparation of a typical ATPS (Esquena, 2016). Upon the vigorous mixing of a PEG/DEX solution, aqueous/aqueous (water-in-water) microdroplets are transiently formed, and various biomolecules and even inorganic materials can be partitioned into, or excluded from, these droplets, due to the chemical and morphological characteristics of the substances partitioned (Akbulut et al., 2012; Jia et al., 2014; Vis et al., 2015). Generally, this behavior of LLPS droplets is sharp (Nott et al., 2016), and could be associated with the origin of life and prebiotic functions (Poudyal et al., 2018). Originally, ATPSs with various hydrophilic polymers, in addition to the above PEG/DEX, have been developed to extract molecules and supramolecules from biological materials (Albertsson, 1971). Their simplicity makes them suitable for harboring functional proteins and living cells. For example, these microdroplets have been used to promote interaction between entrapped proteins to achieve their alignment (Monterroso et al., 2016; Song et al., 2018). Previously, we have reported DNA entrapment (Tsumoto et al., 2015), the formation of microparticles by crosslinking proteins (Tsumoto and Yoshikawa, 2017), and the selective entrapment of filamentous actin (F-actin) and subdomain formation with dsDNAs and F-actin proteins, inside PEG/DEX microdroplets (Nakatani et al., 2018).

ATPS is known as a useful system to get selective partition on various biomacromolecules, DNAs, proteins, etc., under certain compositions of polymers. As has been indicated by Albertsson (1971), previous literatures reveal a large number of phase diagrams for the phase separation of binary or more-component polymer solutions. The ATPS with PEG/DEX is popular in biochemical and biophysical research, and we previously used this system to investigate the dynamics (time-course development) of phase segregation of micro-scale regions (Toyama et al., 2008), as well as the specific localization of DNAs and proteins, such as the cytoskeletal protein actin (Tsumoto et al., 2015; Nakatani et al., 2018). In these studies, we observed aqueous/aqueous (water-in-water) microdroplets with an average diameter of ~10–100 μm. Importantly, the droplets are stably generated and remain after vigorous mixing using a vortex mixer, etc., near the critical point in a phase diagram, because there is only a very small difference in density between the phases containing the two polymers in addition to the weak surface tension, which are unique characteristics of a polymer solution.

Compared to bulk ATPS, the above mentioned aqueous/aqueous microdroplet is suitable for microscopic observation; therefore, we can frequently observe intriguing phenomena with biological macromolecules only when these molecules are encapsulated within such microcompartments. For example, interestingly, actin proteins apparently change their preferred location (distributed evenly, encapsulated within the interior, and adhered to the inner surface) in droplets in PEG/DEX ATPS as their higher-ordered structure changes from monomeric (G-actin) through polymeric (F-actin) to bundled actin (Nakatani et al., 2018). DNAs and F-actin entrapped simultaneously exclude each other to form subdomains inside the droplets. Thus, generally, biopolymers with semi-flexible and rigid chains are usually excluded from the PEG-rich exterior environment to the interior of DEX-rich microdroplets. In addition, further transformation of their structures as well as coexistence with other polymers possessing different properties could cause a change in their own positions.

Considering the unique characteristics to entrap certain biomolecules, it would also be expected that in even simple systems, living cells can find preferred locations in microdroplets and their localization can change with a change in the composition of the ATPS. Cells are much larger than the biomacromolecules, and cellular systems are more complicated. However, even though the model artificial system is quite simple, we would expect it to mimic the migration and arrangement of cells in an aqueous environment inside living bodies in a most basic manner, i.e., without any specific molecular interactions among genetic products.

Recently, Han et al. reported the formation and manipulation of cell spheroids (Han et al., 2015), clearly indicating that ATPS could be a powerful tool for controlling cell position without causing important damage. In the present study, we used two different kinds of cells, erythrocytes (horse red blood cells; RBCs) and NAMRU mouse mammary gland epithelial cells (NMuMG cells) as models, and investigated how these cells are localized when entrapped inside ATPS microdroplets. Interestingly, we found that RBCs and NMuMG cells alternatively preferred to localize either in the interior or at the periphery of microdroplets, and this preference could be switched by changing the PEG/DEX ratio. We considered that the PEG/DEX interface property could change with a change in the interface composition, and this could affect how the interface interacts with different types of cells to cause this switching.

## Materials and Methods

### Reagents

We used polyethylene glycol (PEG) and dextran (DEX) to form an aqueous/aqueous two-phase system (ATPS): PEG 6,000 (molecular weight (M_w_) 7,300–9,300 Da) and DEX 200,000 (M_w_ 180,000–210,000 Da) were purchased from FUJIFILM Wako Pure Chemical Industries (Osaka, Japan). PEG and DEX were stocked as solutions of 20 wt% dissolved in isotonic sodium chloride solution (0.9 wt% solution of NaCl) to regulate the osmotic pressure similar to that of cell membranes. The isotonic sodium chloride solution was prepared with nuclease-free water (Milli-Q, 18.2 MΩ·cm). Other reagents used were of analytical grade.

### Microdroplets

A PEG/DEX solution shows the micro phase segregation at the conditions near to the bimodal line, i.e., the boundary of the homogeneous phase (mono-phase region) to the separated phase (two-phase region) of the solution. Due to the micro phase segregation of polymers, the microdroplets whose diameters range from ~10 to 100 μm could be stable for around several hours after mechanical agitation. In this study, we used the two types of conditions of PEG and DEX concentrations, PEG/DEX = 10%:5%, 5%:5%. Details of experimental solutions used here are described separately in the Supplementary Material.

### Cells

For observations of red blood cells (RBCs), we used the preserved blood of horse purchased from Nippon Bio-test Laboratories, Inc. (Saitama, Japan) and gently added a small aliquot of the blood to the PEG/DEX system without any washing. As model epithelial cells, we used NAMRU mouse mammary gland epithelial cells (NMuMG cells), which were cultivated from cultured cells at regular intervals as previously reported (Yoshida et al., 2017). For mixing in the PEG/DEX system, the epithelial cells were separated in advance from the culture medium by centrifuge.

### Microscopy

We observed ATPS microdroplets using bright field microscopy or confocal laser scanning microscopy (CLSM). Bright field microscopy images were obtained with an Axio Observer.A1 (Carl Zeiss, Germany) equipped with a 40× objective and a CCD digital camera (C11440, Hamamatsu Photonics). CLSM images were acquired with an FV-1000 laser scanning microscope (Olympus, Japan) and the images were analyzed using FV10-ASW software (Olympus). The images acquired with the bright field microscope were analyzed using ImageJ software (Rasband, W.S., ImageJ, US National Institutes of Health, Bethesda, Maryland, USA, http://imagej.nih.gov/ij/, 1997–2016). In the CLSM observation, we used fluorescein isothiocyanate (FITC)-dextran (average M_w_ 250,000, Sigma-Aldrich; Ex. 488 nm) and the lipophilic dye Nile red (Thermo Fisher Scientific; Ex. 543 nm) to fluorescently visualize DEX-rich domains and RBCs, respectively. The details of experimental solutions subjected to the aforesaid observations are described in the Supplementary Material.

## Results and Discussion

**Figure 1:**
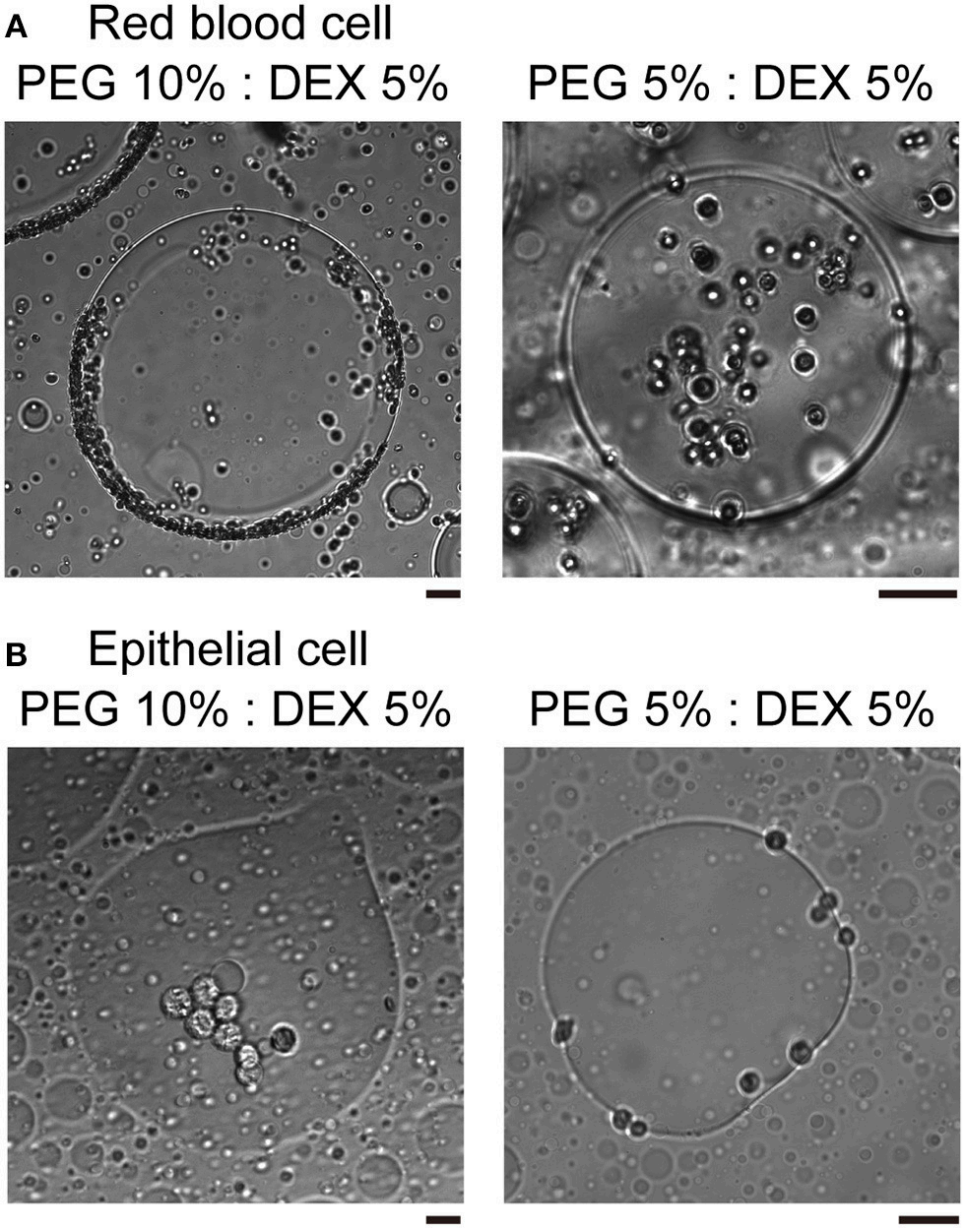
Specific distribution depending on the cell type and the concentration of PEG/DEX. **(A)** Microscopic images of red blood cells (RBCs) in microdroplets for each concentration of PEG/DEX (10%:5%, 5%:5%). The scale bar corresponds to 50 μm. **(B)** Microscopic images of mouse mammary gland epithelial cells (NMuMG cells) in microdroplets for each concentration of PEG/DEX (10%:5%, 5%:5%). Note that the difference in color of cells could be due to a little variation in distance from the focus. Bar: 20 μm.

In the present study, we used two kinds of mammal cells: red blood cells (RBCs) and NMuMG cells, which are a type of epithelial cell. As shown in typical microscopic images in Figure 1, both RBCs and NMuMG cells were entrapped inside DEX-rich microdroplets when they were mixed in the ATPS with PEG/DEX = 10%:5% and 5%:5%. However, a close look at their distribution revealed that their location switched between the center and the periphery of the droplet with a change in the PEG/DEX ratio. In both cases, DEX-rich microdroplets were located on the bottom of the chamber due to their a little bit larger density as observed in the bulk experiment. Interestingly, these cells showed an opposite preferred distribution inside the droplets (Figure 1); at PEG/DEX 10%:5%, RBCs gathered near the interface, while NMuMG cells gathered inside the droplets rather than the interface. In contrast, at PEG/DEX 5%:5%, these preferences were reversed; i.e., RBCs were situated inner part of the droplets, and NMuMG cells were localized at their periphery. RBCs tended not to contact each other when they were trapped inside the droplets at either concentration of the coexisting polymers, whereas NMuMG cells positioned inside the droplets adhered more strongly to each other. Since NMuMG cells are adherent in their intrinsic property, encapsulation within DEX-rich droplets could enhance their attachment propensities (Yoshida et al., 2017).

**Figure 2:**
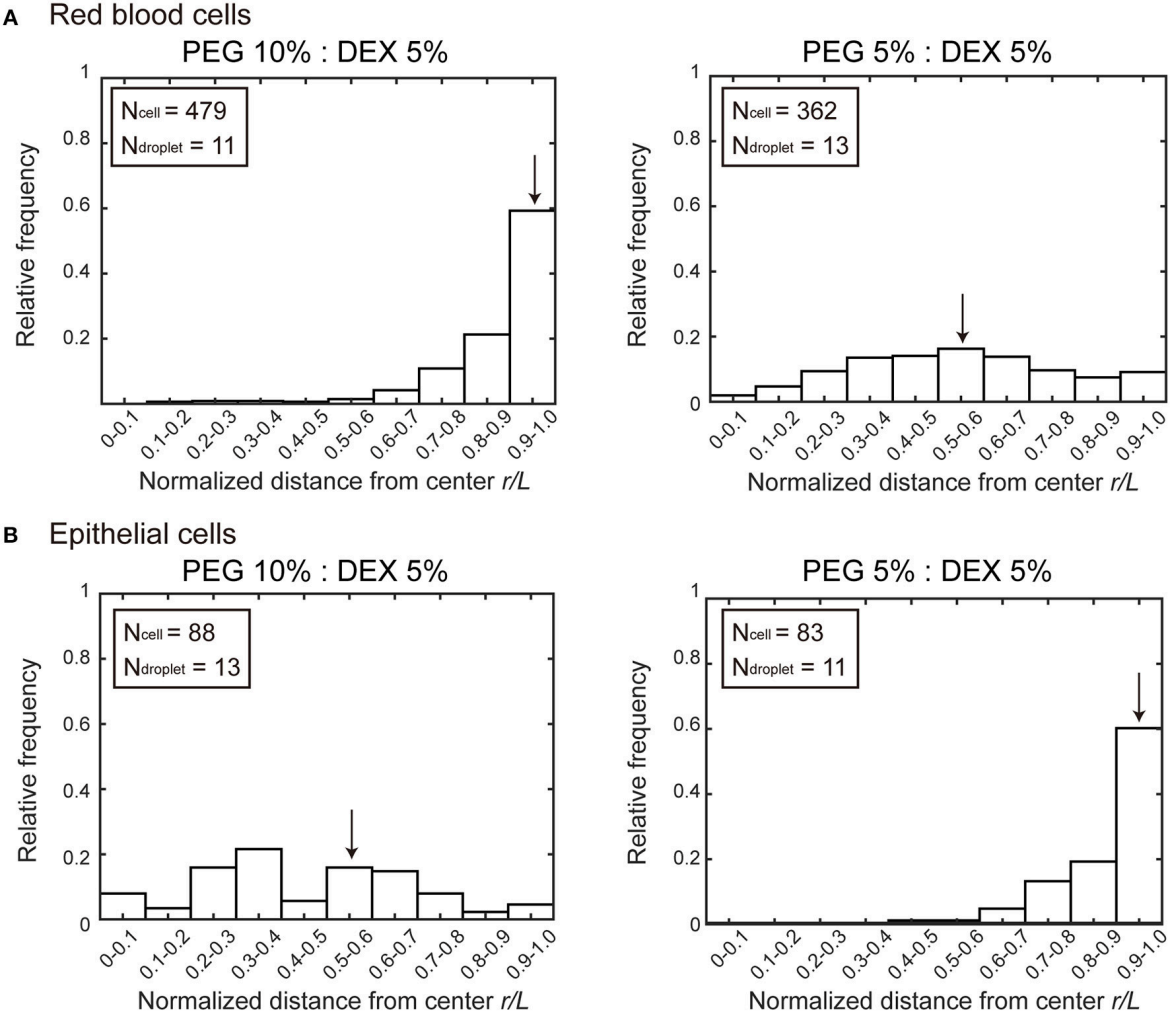
Histograms of positions where **(A)** RBCs and **(B)** NMuMG cells were distributed inside microdroplets with PEG/DEX = 10%:5% and 5%:5%. Horizontal axes indicate normalized distances (*r/L*) from the center of the droplets to the positions of the entrapped cells. The relative radial distance normalized to unity by the average radius *L* of each droplet is 0 at the center and 1.0 at the periphery. The average radius is calculated from the area section of each microdroplet observed by microscopy, the distance *r* at which a cell was observed is divided by *L* of the droplet entrapping the cell to give the normalized radial distance. Some cells were observed at normalized distances greater than unity because some droplets assumed a somewhat aspherical shape, and then the number of these cells are included in the number at *r/L* of 0.9–1.0. Arrows indicate the medians to express the trends in cell localizations. The total numbers of counted cells (N_cell_) and droplets (N_droplet_) are indicated.

Figure 2 shows histograms of the cell population vs. the position of microdroplets. Along the radial distance (*r/L*) normalized to unity by the average radius *L* of each droplet, entrapped cells were counted and the numbers were converted into a cell frequency. In both types of cells, the median location, which is indicated by arrow, of entrapped cells shifted from around the center (relative radial distance *r/L* ~0.5) to the periphery (relative radial distance *r/L* 1.0). But the preference is opposite; i.e., RBCs and NMuMG cells (epithelial cells) were observed adsorbed to the interfaces at PEG/DEX = 10%:5% and 5%:5%, respectively. It should be noted that if cells were included inside a droplet without any attraction but with some repulsion to the interface, a peak of the population may appear around *r/L* ~0.5 because of the larger area compared to the region near the center.

**Figure 3:**
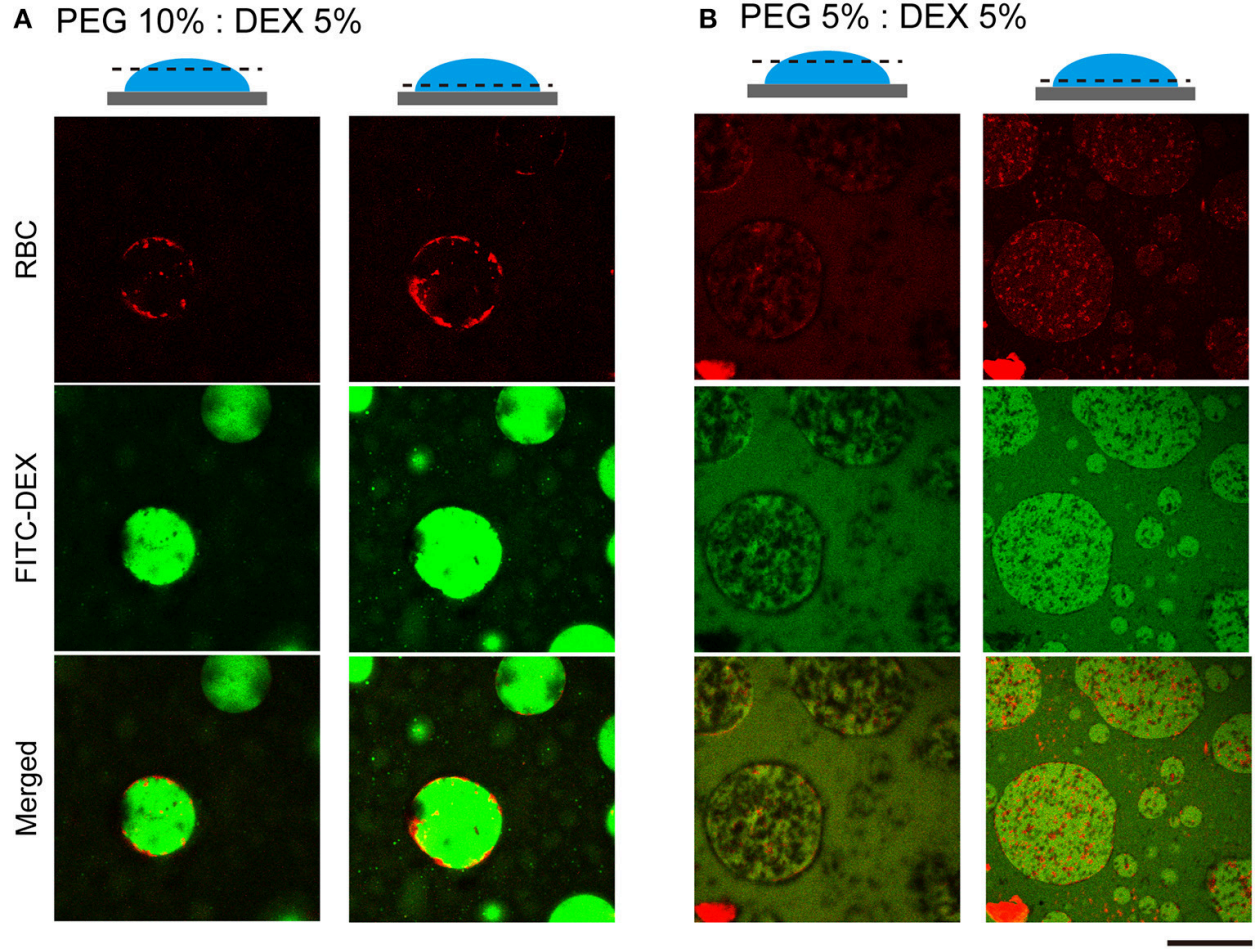
Confocal laser scanning microscopy (CLSM) images of RBCs in microdroplets. Top: RBCs labeled with Nile red. Middle: FITC-DEX. Bottom: Merged. The focal planes of the left and right columns for each condition are around the top and below the middle, respectively, as schematically illustrated for PEG/DEX = **(A)** 10%: 5% and **(B)** 5%: 5%. Bar: 100 μm.

To further examine how cells switched their distribution inside the droplets, we conducted CLSM observation of the PEG/DEX ATPS containing RBCs labeled fluorescently by Nile red (Figure 3). FITC-DEX illuminated the microdroplets and RBCs emitting red fluorescence were fully entrapped, but their distribution was apparently different at PEG/DEX = 10%:5% and 5%:5%, as in Figure 1. Under the former condition, RBCs were visualized on the inner interface of the DEX-rich droplet emitting green fluorescence, and in the latter, RBCs were relatively dispersed into the DEX-rich droplets. Fewer cells were observed at the top than the middle of the droplet, indicating that entrapped cells could fall under the present conditions. A number of RBCs were here also found at the interface of the microdroplet at PEG/DEX = 5%/5% (Figure 3B), and this trend is consistent with the localization shown in Figure 2A.

**Figure 4:**
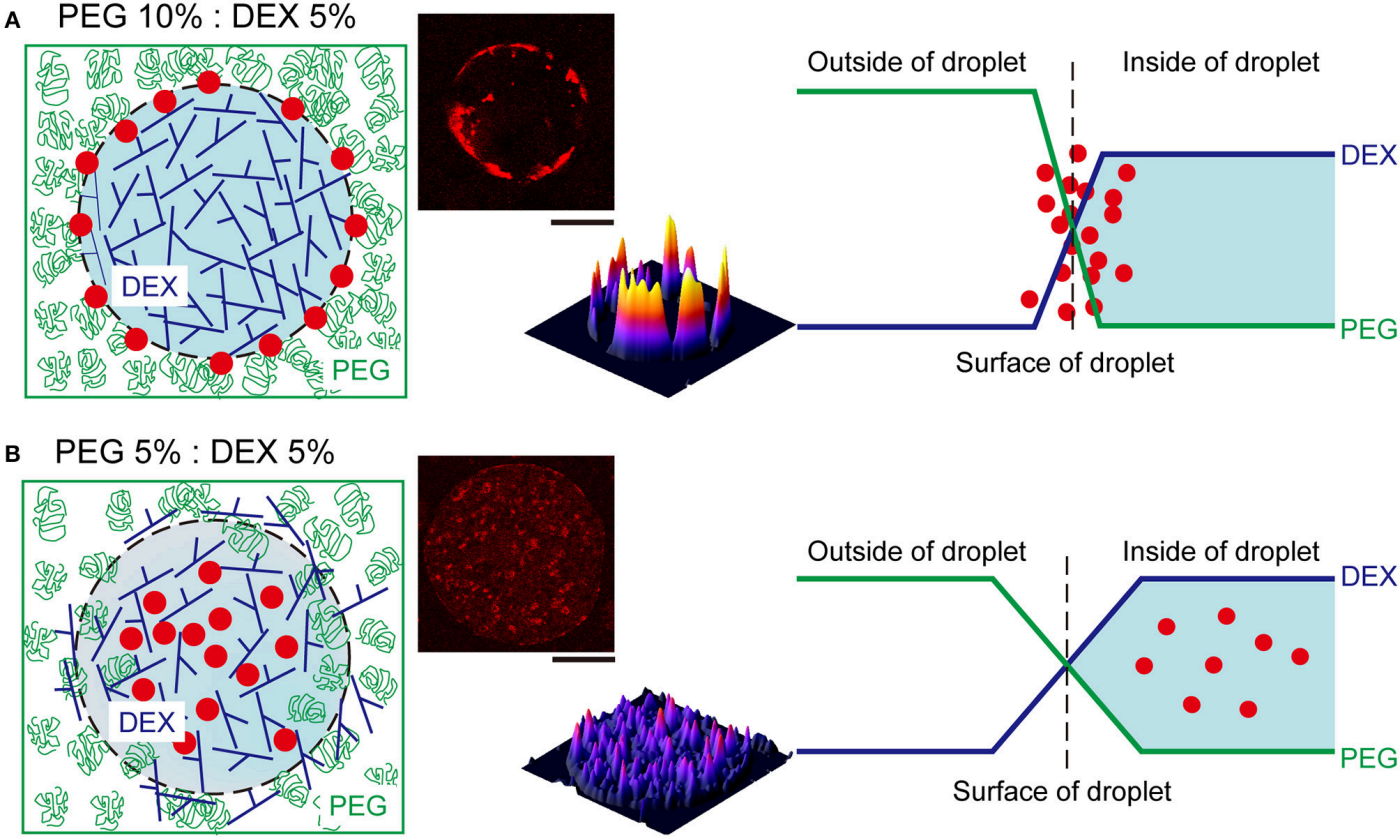
Schematic representation of the proposed mechanism of RBC distribution inside microdroplets generated by PEG and DEX. The leftmost illustrations show localization of RBCs (red closed circles) in droplets of DEX (blue branching line) surrounded with PEG (green random coil). The rightmost graphs depict the profiles of concentrations of DEX and PEG at the interfaces of droplets. **(A)** At PEG/DEX = 10%: 5%, the high surface energy due to the steep gradient of polymer concentrations may cause the accumulation of RBCs at the interface to lower the tension. **(B)** In contrast, at PEG/DEX = 5%: 5%, the surface tension can be moderately reduced to permit mutual invasion to some extent, and thus RBCs cannot be pushed to the interface so strongly. The pseudo three-dimensional images in the middle show the fluorescence intensities of Nile red from RBCs entrapped inside the DEX-rich microdroplets shown in the upper-left side, also in each upper-right panel of Figure 3. Bar: 100 μm.

We here observed the simple switching of cell localization inside DEX-rich droplets. Both cell types, RBCs and NMuMG cells, preferred the interior, but they exhibited opposite trends when settling in different compartments. The preference in cell localization may be caused by various factors related to difference in cell properties, including their sizes, morphologies, surface structures, adhesive (non-adhesive) behaviors, number density, etc., so it is too complicated to permit us to give a detailed precise explanation for the mechanism. Therefore, the present condition we investigated is considered to be moderately suitable for simple demonstration of switchable behavior of cell localization inside ATPS droplets. For speculation, instead, we try to simply discuss this different preference by imagining cells with different dimensions under a crowded environment with PEG/DEX schematically illustrated in Figure 4 as follows.

Generally, DEX molecules have branched structures with some room for harboring other biomacromolecules, and PEG molecules have linear flexible structures with a high excluded volume under concentrated conditions. Since these polymers provide different aqueous environments, not only large biomolecules but also cells prefer the DEX-rich phase, which is less crowded than the PEG-rich phase. In the present study, we used two PEG/DEX ATPSs with different conditions; one is near the critical point in the phase diagram (5%:5%); the other is somewhat far from that point, and accordingly, the binodal line (10%:5%).

The surface tension with such aqueous/aqueous interfaces is not so strong compared to oil/water interfaces (Atefi et al., 2014). Therefore, it can be finely modulated by changing the PEG/DEX ratio. In the present case, at PEG/DEX = 10%:5%, the interface has a higher tension (surface energy) with a steeper gradient of polymer concentrations, and thus RBCs, which are relatively smaller than NMuMG cells, could keep located on the surface, leading to reduction of the surface energy (Figure 4A). On the other hand, at PEG/DEX = 5%:5%, the surface free energy, or surface tension, is so low due to a small gradient of polymer concentrations that small RBCs with weaker effects on stabilization of the interface alternatively tend to leave and enter the interior of DEX-rich droplets, which can accommodate these cells (Figure 4B).

To interpret the opposite preference of NMuMG cells along the same scenario as the case of RBCs, their larger size can be taken into account in relation to depletion effects by PEG. NMuMG cells appear to move to the interior of DEX-rich droplets, avoiding the interface where PEG exists more densely at PEG/DEX = 10%:5%. In other words, PEG could effectively induce depletion effect on NMuMG cells due to their larger sizes (surface areas), so these cells can be located not near a PEG-rich surrounding but in a DEX-rich inner space, where they might be associated with each other by moderate depletion force with DEX polymers (Atefi et al., 2015). At PEG/DEX = 5%:5%, where most RBCs are located inside the droplets, NMuMG cells can stand adhering to the interface, leading to the efficient stabilization due to their greater dimension. Note that the surface free energy of the interface through the phase segregation of polymer solutions is generally much smaller than those for interfaces with small molecules, because of the small contribution of the mixing entropy for polymer systems (Nakatani et al., 2018).

It has been shown that interface properties are in general strongly correlated to the positioning and qualities of cells with various origins (Atefi et al., 2015; Han et al., 2015) with some changes in genetic activities (Yu et al., 2018). To achieve assembly of highly organized compartments including cells in microfluidics, interfacial environments where cells and biopolymers are accumulated should be well-regulated (Yamada et al., 2016). We here simply chose physiological saline for suspending cells, but it is not suitable for cell proliferation, but, along this line, when some conditions that facilitate micro phase separation and cell growth are available, our results would be expected to aid development of such techniques for constituting assembled cell systems.

In conclusion, it was found that living cells exhibit specific localization in an aqueous solution with water/water micro-droplets generated through phase separation in the presence of hydrophilic binary polymers. It was revealed that two type of cells are situated either in the interior or at the periphery of the droplets. By changing the relative ratio of the polymer content, switching of the cell localization was observed. The mechanism of such observations were interpreted in terms of polymer depletion effect on the micro phase separation. It is highly expected that the simple mixing procedure with micro water/water droplets provides novel methodology to construct 3D cellular assembly composed with difference cell species.

## Author Contributions

HS and TF conducted the experiments with the help of KT under direction by KY. HS, TF, and KT wrote the manuscript with the help of YY and YH under supervision by KY.

### Conflict of Interest Statement

The authors declare that the research was conducted in the absence of any commercial or financial relationships that could be construed as a potential conflict of interest.
