# Supplementary material for: Aqueous/Aqueous Micro Phase Separation: Construction of an Artificial Model of Cellular Assembly
Source: Front Chem. 2019 Feb 1;7:44. doi: 10.3389/fchem.2019.00044 (PMC6367680; doi:10.3389/fchem.2019.00044)
Supplement: Supplementary file 1 [file Data_Sheet_1.PDF]

Supplementary Material

Aqueous/Aqueous Micro Phase Separation: Construction of an Artificial Model of Cellular Assembly

Hiroki Sakuta, Tadashi Fujimoto, Yusuke Yamana, Yusuke Hoda, Kanta Tsumoto*, Kenichi Yoshikawa

*** Correspondence:** Kanta Tsumoto: tsumoto@chem.mie-u.ac.jp

1 Supplementary tables of solution details

The detailed compositions of the experimental solutions which were in turn subjected to microscopic observations are described in the following subsections. Each portion from the stock solutions or cell suspensions was added in the order indicated in the Tables unless other conditions were mentioned.

1.1 Solution details of experiment (Figures 1 and 2)

Table S1. Experimental solution for *Figures 1 and 2 (A) Red blood cells at PEG 10% : DEX 5%*

Components	Stock solution	Volume added (µL)	Final concentration
PEG aq.*	20 wt%	50 µL	10 %
DEX aq.*	20 wt%	25 µL	5.0 %
NaCl aq.	0.9 wt%	23µL	0.9 %
Red blood cell	1×	2 µL	1/50×
	Total volume	100 µL	

Table S2. Experimental solution for *Figures 1 and 2 (A) Red blood cells at PEG 5% : DEX 5%*

Components	Stock solution	Volume added (µL)	Final concentration
PEG aq.*	20 wt%	25 µL	5.0 %
DEX aq.*	20 wt%	25 µL	5.0 %
NaCl aq.	0.9 wt%	48 µL	0.9 %
Red blood cell	1×	2 µL	1/50×
	Total volume	100 µL	

Table S3. Experimental solution for *Figures 1 and 2 (B) Epithelial cells at PEG 10% : DEX 5%*

Components	Stock solution	Volume added (μL)	Final concentration
PEG aq.*	20 wt%	250 μL	10 %
DEX aq.*	20 wt%	250 μL	5.0 %
NaCl aq.	0.9 wt%	500 μL	0.9 %
Epithelial cell**	-		-
	Total volume	1000 μL	

Table S4. Experimental solution for *Figures 1 and 2 (B) Epithelial cells at PEG 5% : DEX 5%*

Components	Stock solution	Volume added (μL)	Final concentration
PEG aq.*	20 wt%	250 μL	5.0 %
DEX aq.*	20 wt%	250 μL	5.0 %
NaCl aq.	0.9 wt%	500 μL	0.9 %
Epithelial cell**	-		-
	Total volume	1000 μL	

* PEG aq. and DEX aq. were dissolved with isotonic sodium chloride solution (0.9 wt% NaCl aq.).

** Epithelial cells (NAMRU mouse mammary gland epithelial cells, NMuMG cells) were cultivated from cultured cells at regular intervals as previously reported (ref. Yoshida et al., 2017) and, they were suspended with PEG, DEX and NaCl solutions after centrifugation and removal of the culture media.

(Ref.)

Yoshida, A., Tsuji, S., Taniguchi, H., Kenmotsu, T., Sadakane, K., and Yoshikawa, K. (2017) Manipulating living cells to construct a 3D single-cell assembly without an artificial scaffold. *Polymers* 9, 319. doi:10.3390/polym9080319

1.2 Solution details of experiment (Figure 3)

Table S5. Experimental solution for *Figure 3 (A) Red blood cells at PEG 10% : DEX 5%*

Components	Stock solution	Volume added (µL)	Final concentration
PEG aq.*	20 wt%	50 µL	10 %
DEX aq.* (FITC-DEX**)	20 wt%	25 µL	5.0 %
NaCl aq.	0.9 wt%	16 µL	0.9 %
Red blood cell	1×	1 µL	1/100×
Nile red***	520 µM	8 µL	41.6 µM
	Total volume	100 µL	

Table S6. Experimental solution for *Figure 3 (B) Red blood cells at PEG 5% : DEX 5%*

Components	Stock solution	Volume added (µL)	Final concentration
PEG aq.*	20 wt%	25 µL	5.0 %
DEX aq.* (FITC-DEX**)	20 wt%	25 µL	5.0 %
NaCl aq.	0.9 wt%	41 µL	0.9 %
Red blood cell	1×	1 µL	1/100×
**Nile red*****	520 µM	8 µL	41.6 µM
	Total volume	100 µL	

* PEG aq. and DEX aq. were dissolved with isotonic sodium chloride solution (0.9 wt% NaCl aq.).

** Fluorescent-labeled DEX (fluorescein isothiocyanate (FITC)-dextran, Sigma Aldrich, average M_w = 250,000) was contained with 5 % of the total amount of DEX. The final ratio of FITC-DEX was approximately 0.25 %.

*** Nile red was dissolved in ethanol at 520 µM for the stock solution.
